# Light Regulates the RUBylation Levels of Individual Cullin Proteins in *Arabidopsis thaliana*

**DOI:** 10.1007/s11105-017-1064-9

**Published:** 2018-01-29

**Authors:** Matthew J. Christians, Aron Rottier, Carly Wiersma

**Affiliations:** Department of Cell and Molecular Biology, Grand Valley State University, 3300A Douglas Kindschi Hall of Science, Allendale, MI 49401 USA

**Keywords:** Cullin, NEDD8, Photomorphogenesis, Phytochrome, RUB, Ubiquitin

## Abstract

In plants, the small protein related to ubiquitin (RUB) modifies cullin (CUL) proteins in ubiquitin E3 ligases to allow for efficient transfer of ubiquitin to substrate proteins for degradation by the 26S proteasome. At the molecular level, the conjugation of RUB to individual CUL proteins is transient in nature, which aids in the stability of the cullins and adaptor proteins. Many changes in cellular processes occur within the plant upon exposure to light, including well-documented changes in the stability of individual proteins. However, overall activity of E3 ligases between dark- and light-grown seedlings has not been assessed in plants. In order to understand more about the activity of the protein degradation pathway, overall levels of RUB-modified CULs were measured in *Arabidopsis thaliana* seedlings growing in different light conditions. We found that light influenced the global levels of RUBylation on CULs, but not uniformly. Blue light had little effect on both Cul1 and Cul3 RUBylation levels. However, red light directed the increase in Cul3 RUBylation levels, but not Cul1. This red-light regulation of Cul3 was at least partially dependent on the activation of the phytochrome B signaling pathway. The results indicate that the RUBylation levels on individual CULs change in response to different light conditions, which enable plants to fine-tune their growth and development to the various light environments.

## Introduction

The ubiquitin/proteasome system (UPS) serves as an important mechanism for selectively targeting proteins for breakdown in eukaryotic cells, where small polymeric chains of the 76-amino-acid ubiquitin (Ub) protein are covalently ligated to a lysine residue on a substrate protein (Hershko and Ciechanover 1998). These ubiquitylated substrates are then recognized by the 19S regulatory lid of the 26S proteasome and subsequently degraded by tryptic catalysis in the proteasome core (Voges et al. 1999; Thrower et al. 2000). The attachment of Ub to select substrates is accomplished through a three enzyme (E1-E2-E3) cascade, where an E1 enzyme activates Ub in an ATP-dependent manner, Ub is then transferred to an E2-conjugating enzyme, and the E3-ligase couples the Ub to the substrate (Pickart 2001). There is a vast assortment of ubiquitylated substrates, which is attained through an almost equally vast array of E3 ligase substrate adaptors. In *Arabidopsis thaliana*, for example, there are over 1500 substrate adaptor genes, many of which have been characterized, and play key roles in numerous plant growth and developmental regimens. These include responses to biotic and abiotic factors, such as hormone, pathogen, and light responses (Moon et al. 2004; Vierstra 2009; Choi et al. 2014).

The cullin-RING ligases (CRLs) comprise the bulk of the E3 ligase family in plants (estimated at 863 CRL genes in *A. thaliana* and 991 in *O. sativa*) (Hua and Vierstra 2011). These multi-subunit complexes consist of a cullin (CUL) backbone subunit, which positions the substrate in close proximity to the E2-Ub and allows the transfer of Ub to the substrate protein. The substrate is recruited to the E3 ligase by way of unique adaptor proteins which assemble on the amino-terminal end of cullin, while the RING-box (RBX)-1 subunit attaches to the carboxy-terminal end and brings in the E2-Ub (Zheng et al. 2002b; Sarikas et al. 2011). There are several substrate adaptor protein families in *Arabidopsis*, each assembled onto their cognate CULs. Adaptors containing F-box domains assemble with Cul1/2 through an additional ASK1/2 linker protein. The broad complex/tramtrack/bric-a-brac (BTB) proteins assemble directly with Cul3a and b, while DWD motif proteins assemble with Cul4 through an additional DDB1 protein adaptor (Risseeuw et al. 2003; Dieterle et al. 2005; Gingerich et al. 2005; Lee et al. 2008).

The assembly of the substrate/adaptor/cullin/RBX/E2-Ub complex alone is not enough for ubiquitylation to occur. Related to ubiquitin (RUB), also known as Nedd8 (neural precursor cell, developmentally downregulated 8) in animals, activates the E3 complex. As its name suggests, RUB closely resembles Ub in sequence, structure, and function. It uses a separate but similarly fashioned E1–E2–E3 biochemical pathway for activation, conjugation, and ligation. However, CULs are the primary target for RUB modification (Mergner and Schwechheimer 2014). Several studies suggest that RUB serves as part of the docking site for the E2-Ub, and RUBylation initiates a conformational shift in CUL to facilitate the transfer of Ub from the E2 to the substrate (Kawakami et al. 2001; Sakata et al. 2007; Duda et al. 2008).

Although RUB is required for E3 ligase activity, constitutive RUBylation of CUL causes unnecessary ubiquitylation and degradation of E3 ligase components, including the substrate adaptors and CULs (Wu et al. 2005; Schmidt et al. 2009). To avoid this, CUL cycles through RUB-modified and unmodified states. DeRUBylation occurs through the activity of the COP-9-signalosome (CSN), while the cullin-associated Nedd8 dissassociation-1 (CAND1) protein can keep CUL in the unRUBylated form by blocking the RUBylation site. When no substrate/adaptor is present, CAND1 and the CSN are actively promoting the De-RUBylated state of CULs (Lyapina et al. 2001; Zheng et al. 2002a; Mergner and Schwechheimer 2014). This cycling is also thought to increase the dynamic capabilities of the UPS by maintaining a pool of free CUL for new substrate adaptors to bind.

In plants, a robust connection between defects in the RUBylation pathway and different developmental responses has been established. *Arabidopsis* plants containing mutations in the E1-activating enzyme (AXR1), E2-conjugating enzyme (RCE1), RBX1, CAND1, and RUB1 and 2 all show auxin related developmental defects in seedlings (Pozo et al. 1998; Dharmasiri et al. 2003; Cheng et al. 2004; Bostick et al. 2004). CSN mutants are constitutively photomorphogenic (Wei and Deng 2003). However, the application of auxin does not seem to affect the RUBylation level of AtCul1 in wild-type seedlings, and little is known to what extent (if any) RUBylation levels are regulated in response to different physiological conditions in plants, including how plants grow in response to light, also known as photomorphogenesis (Pozo et al. 1998, 2002).

Light controls numerous plant developmental responses, including seed germination, circadian rhythms, shade avoidance, chloroplast development, flowering, and senescence. Plants harbor several different photoreceptors to detect incoming light, including UV-B by the UVR8 protein, UV-A/blue light (B) by the cryptochromes, phototropins, and LOV domain-containing proteins, and red/far-red (R/FR) light by the phytochromes (Kami et al. 2010; Jenkins 2014). Each family of photoreceptors initiates unique signaling pathways which lead to appropriate physiological changes, and protein degradation plays a central role in the regulation of each pathway.

The transition from skotomorphogenesis (dark-grown) to photomorphogenesis leads to massive transformations in morphology and physiology in plants, and the widespread alterations in transcription and translation mechanisms that coordinate these changes are still being studied (Casal and Yanovsky 2004; Liu et al. 2012). Changes in selective protein degradation related to this transition have been extensively documented through the assessment of ubiquitylation and stability of individual proteins. Attempts to study global changes in protein degradation in photomorphogenesis have begun using proteomic tools involving mass spectrometry (Aguilar-Hernández et al. 2017). Yet, to our knowledge, the general activity of E3 ligases in dark and light-grown plants has not been assessed. Therefore, we investigated the RUBylation levels of two different cullins (AtCul1 and AtCul3) under different continuous light conditions in *Arabidopsis thaliana*. We found overall RUBylation of CULs increased in response to white light and, surprisingly, distinct RUBylation changes on Cul1 and Cul3 in response to red light. This suggests that overall RUB-CUL formation, and thus active protein degradation, changes in response to light conditions, and the regulation of RUBylation varies between individual CUL proteins.

## Materials and Methods

### Plant Growth Conditions

All seeds were surface sterilized with 25% bleach, then stratified for at least 4 days in darkness at 4 °C to promote germination. Col-0 and HA-Strep-Nedd8 (HSN) seeds were plated on Murishige and Skoog (MS) basal salt media (pH 5.6) (Sigma), supplemented with 2% sucrose and 0.7% agar, and overlaid with a rehydrated cellulose membrane (Research Products International). Seeds were incubated at 21 °C in white light for 16 h to induce germination and then exposed to various continuous light treatments. Induction of HA-Strep-Nedd8 protein in HSN seedlings was performed according to Hakenjos et al. (2011). Briefly, HSN seedlings were treated with liquid MS media (pH 5.6) supplemented with 2% sucrose and 30 μM dexamethasone (Sigma) or ethanol (control) for 15 h before sample collection. Seedlings were harvested and frozen in liquid nitrogen. For the light treatments, white light (W) was supplied by F39T5 841 HO fluorescent mercury bulbs (Philips, USA). R (660 nm), FR (740 nm), and B (450 nm) were supplied by the Z series LED bulbs (HiPoint Corp., Taiwan).

### Immunoblot Analysis

Proteins were extracted from 4-day-old seedlings grown in darkness or in 56 μMol m^−2^ s^−1^ white light (W), 50 μMol m^−2^ s^−1^ R, 10 μMol m^−2^ s^−1^ B, or 10 μMol m^−2^ s^−1^ FR light for general light experiments, and 5, 20, 50, and 100 μMol m^−2^ s^−1^ for R light intensity experiments. Proteins were extracted according to the procedure in Christians et al. (2012) with minor changes. Briefly, seedlings were boiled in 2× extraction buffer (100 mM MOPS, 50 mM NaMetabisulfite, 2% SDS, 20% glycerol, 4 mM EDTA, 10% 2-mercapthoethanol) for 10 min, after which they were homogenized. The solution was then centrifuged at 12,000×*g* for 5 min, and the supernatant was either directly subjected to SDS-PAGE, or total protein concentration was determined by the bicinchoninic acid (BCA) assay (Pierce) before subjected to SDS-PAGE analysis. Ten percent polyacrylamide gels were used to ensure adequate separation of CUL, RUB-CUL, and HSN-CUL proteins. Proteins were transferred to PVDF membrane (LICOR) by electrophoretic transfer. CULs were detected with anti-Cul3 (BML-PW0470) and anti-Cul1 (BML-PWO190) antibodies (Enzo Life Science). RUB and HSN were detected with anti-Nedd8 (Ab205201, Abcam) and anti-HA (715500, Life Technologies) antibodies, respectively. The 20S proteasome subunit PBA1 (*At4g31300*) was detected by anti-PBA1 antibody obtained from Dr. Richard Vierstra’s lab (Yang et al. 2004). Signal detection was performed using near-infrared fluorescently-labeled secondary antibodies (IRDye) on an Odyssey Fc imaging system (LICOR). For quantification, the ratio of RUBylated-Cul to unRUBylated-Cul or the fold-change (light/Dk or mutant/Col-0) of RUB-CUL and PBA1 were calculated from at least three independent replicates subjected to SDS-PAGE. Band intensities were measured using the Odyssey Fc imaging system software.

## Results

### Light Regulates Total RUBylation Levels of CULs

**Fig. 1:**
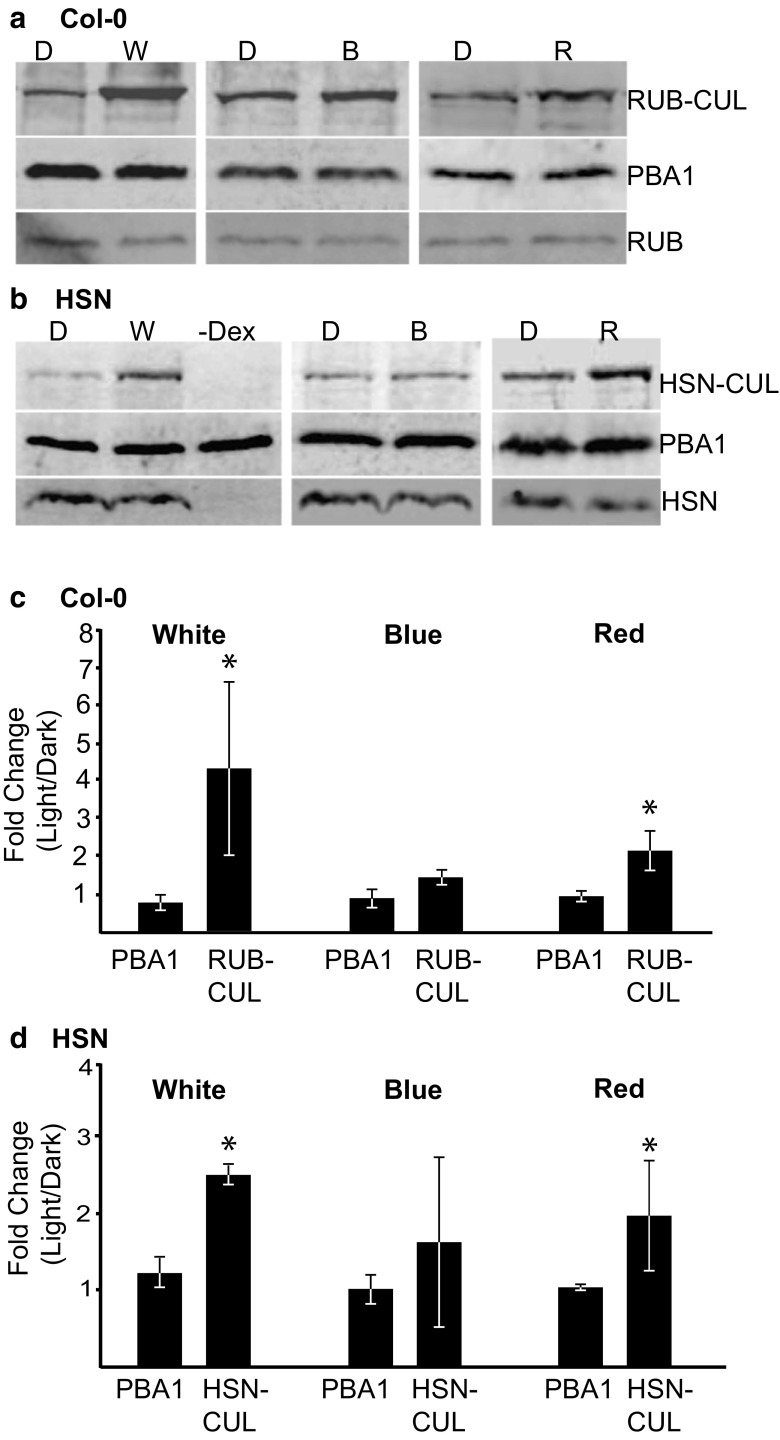
The RUBylation level of CULs increase in light. **a**, **b** Immunoblot analysis of total protein extracts from 4-day-old Col-0 or HSN seedlings grown in Dk, W (56 μMol m^−2^ s^−1^), B (10 μMol m^−2^ s^−1^) or R (50 μMol m^−2^ s^−1^). HSN–Dex seedlings were grown in W. Total RUB and RUB-CUL or HSN and HSN-CUL were detected with anti-Nedd8 or anti-HA antibody respectively. PBA1 was used as a loading control. **c, d** Quantification of the relative change in band intensity in light vs Dk for PBA1 or RUB-CUL displayed as a ratio of light (W, B, or R)/Dk. Asterisks identify significant differences in the fold change between PBA1 and the RUB/HSN-CUL (*p* ≤ 0.05, Student’s *T* test). Error bars represent standard deviation (*n* = 3)

Since RUB shares 83% identity to Nedd8 in humans (Mergner and Schwechheimer 2014), we were able to use an anti-Nedd8 antibody (against human Nedd8) to identify unconjugated RUB and RUB-conjugated CULs in *Arabidopsis* wild-type plants. Unconjugated RUB was detected at 8 kDa, and we detected an intense band at roughly 95 kDa (Fig. 1a), which is near the predicted size of most RUB-CULs (www.arabidopsis.org). To confirm the patterns of RUBylation in the wild-type plants, we also performed the same experiments with plants containing a dual HA-Strep-tagged version of RUB (HA-Strep-Nedd8, HSN) driven by a dexamethasone-inducible system (Hakenjos et al. 2011). Unconjugated HSN and HSN-CUL were detected with anti-HA antibody near 15 and 105 kDa respectively (Fig. 1b).

As photomorphogenesis induces large-scale changes within the plant, we investigated the overall RUBylation status of CULs in plants grown in darkness or different light environments. Plants grown in continuous white light (W) had similar levels of unconjugated RUB, but an increased level of total RUB-CUL compared to dark (D) controls for both Col-0 and HSN plants (Fig. 1). To distinguish if this difference is due to a particular light signaling pathway, we grew plants in continuous blue (B) light (10 μMol m^−2^ s^−1^) and red (R) light (50 μMol m^−2^ s^−1^). In Col-0 and HSN, both B and R increased total RUB-CUL levels, but not to the extent of W (Fig. 1). Quantification of the relative change in band intensities in light compared to dark confirms our observations that RUB-CUL increases in response to multiple light conditions, but the control, 20S proteasome subunit PBA1, does not (Fig. 1c, d). Together, these findings suggest that there is an overall increase in activation of CUL-based E3 ligases in response to light in developing seedlings.

### RUBylation of AtCul1 and AtCul3 Are Differentially Regulated by Light

**Fig. 2:**
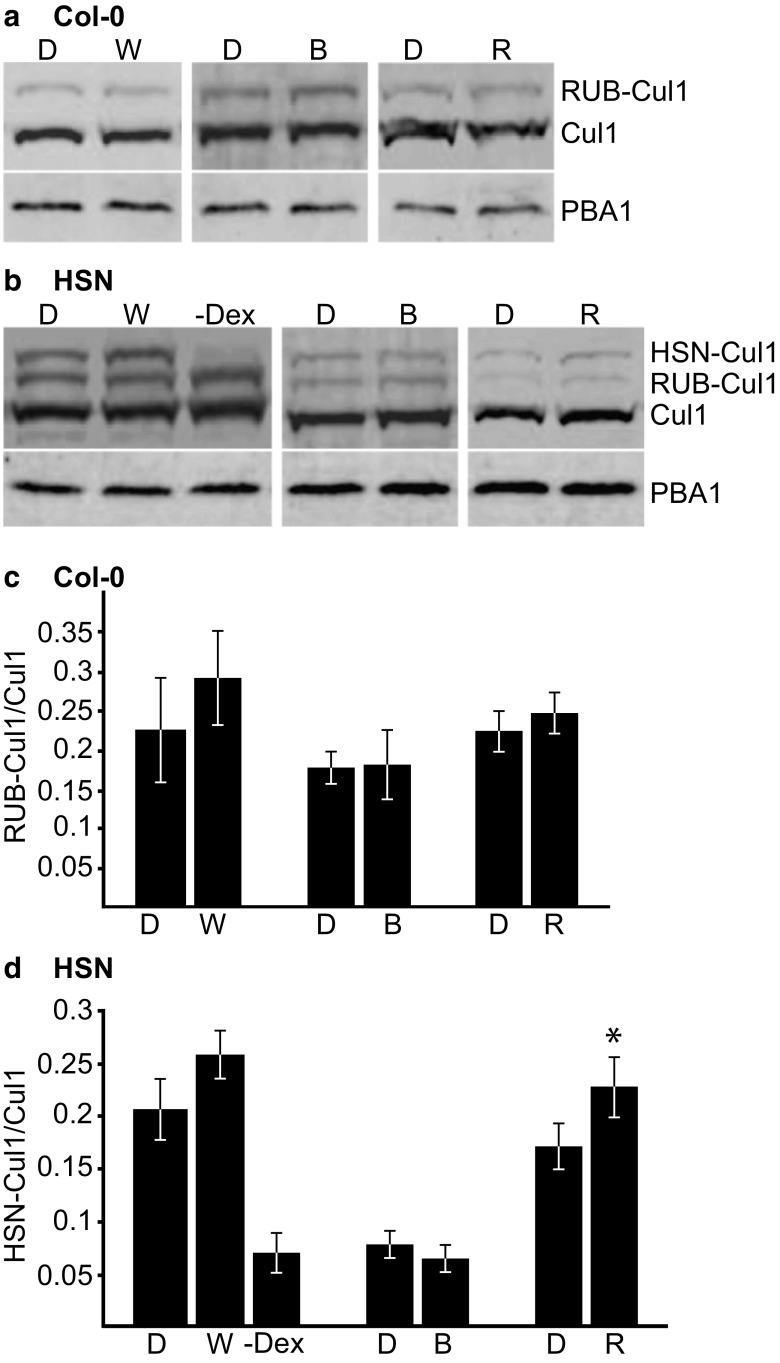
The RUBylation level of AtCul1 in response to light. **a**, **b** Immunoblot analysis with anti-Cul1 antibody of total protein extracts from 4-day-old Col-0 or HSN seedlings grown in Dk, W (56 μMol m^−2^ s^−1^), B (10 μMol m^−2^ s^−1^), or R (50 μMol m^−2^ s^−1^). HSN–Dex seedlings were grown in W. PBA1 was used as a loading control. **c**, **d** Quantification of RUB-Cul1 or HSN-Cul1 levels displayed as a ratio of RUB-Cul1 or HSN-Cul1 to unmodified Cul1 levels. Asterisks identify significant differences between D and light samples (*p* ≤ 0.05, Student’s *T* test). Error bars represent standard deviation (*n* = 3)

Since total RUBylation levels of CULs are regulated by light, we wanted to develop a more detailed analysis of which CULs may participate in this type of regulation. To do this, the RUBylation levels of two CULs (AtCul1 and AtCul3) were analyzed in response to W, B, and R. Using anti-AtCul1 or anti-AtCul3 antibody, two bands were detected in Col-0 and three bands in HSN seedlings that correspond to unmodified Cul1/3 (85 kDa), RUB-modified Cul1/3 (95 kDa), and HSN-modified Cul1/3 (105 kDa). The RUBylation levels of AtCul1 in both Col-0 and HSN seedlings did not significantly change much in response to most light conditions tested compared to the D controls. Only in HSN seedlings grown in R do we see slightly higher levels that are significant. These results indicate that these light conditions do not lead to a large increase in activation of AtCul1-containing E3 ligase complexes (Fig. 2).

**Fig. 3:**
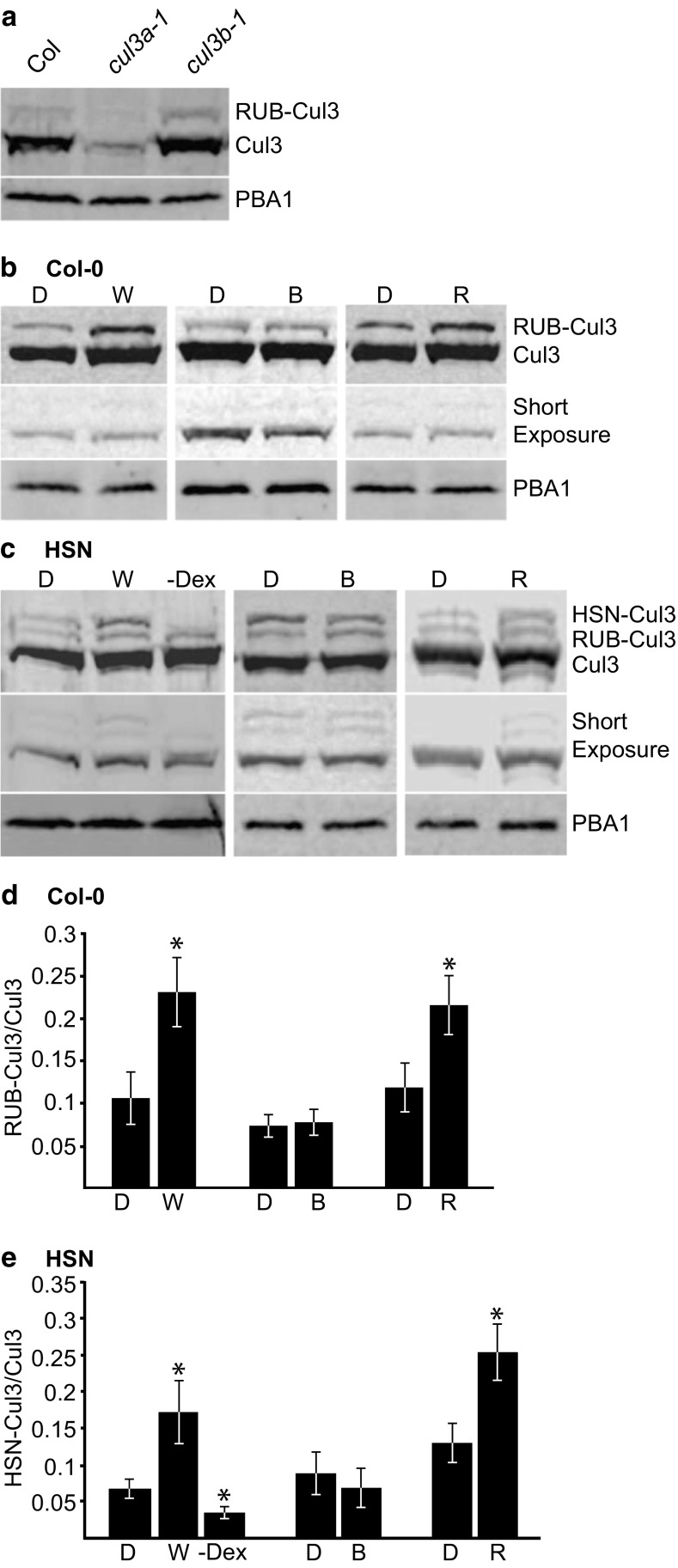
The RUBylation level of Cul3 increases in W and R light. Immunoblot analysis of total protein extracts from 4-day-old seedlings with anti-Cul3 antibody. **a** Col-0, *cul3a-1*, and *cul3b-1* were exposed to R (50 μMol m^−2^ s^−1^). PBA1 was used as a loading control. **b**, **c** Col-0 or HSN seedlings grown in Dk, W (56 μMol m^−2^ s^−1^), B (10 μMol m^−2^ s^−1^), or R (50 μMol m^−2^ s^−1^). HSN–Dex seedlings were grown in W. PBA1 was used as a loading control. The short exposure shows the relative levels of unmodified Cul3. **d**, **e** Quantification of RUB-Cul3 or HSN-Cul3 levels displayed as a ratio of RUB-Cul3 or HSN-Cul3 to unmodified Cul3 levels. Asterisks identify significant differences between Dk and light samples (*p* ≤ 0.05, Student’s *T* test). Error bars represent standard deviation (*n* = 3)

Given that two isoforms of Cul3 are present in *Arabidopsis thaliana* and they share 88% identity, we wanted to determine the specificity of the Cul3 antibody to Cul3a and Cul3b. *Arabidopsis* mutants *cul3a-1* and *cul3b-1* contain null mutations in *CUL3A* and *CUL3B* genes, respectively, which result in undetectable levels of mRNA transcript (Gingerich et al. 2005). Upon immunoblot analysis with Cul3 antibody, a faint band was detected in *cul3a-1* at 95 kDa, which most likely represents Cul3b, while there was a strong band in *cul3b-1*, which most likely represents Cul3a (Fig. 3a). This suggests that either the Cul3 antibody recognizes Cul3a at a higher affinity than Cul3b or that Cul3a is expressed at a substantially higher level than Cul3b. Regardless of which scenario is true, it seems that we are detecting both isoforms, the majority of which is Cul3a.

**Fig. 4:**
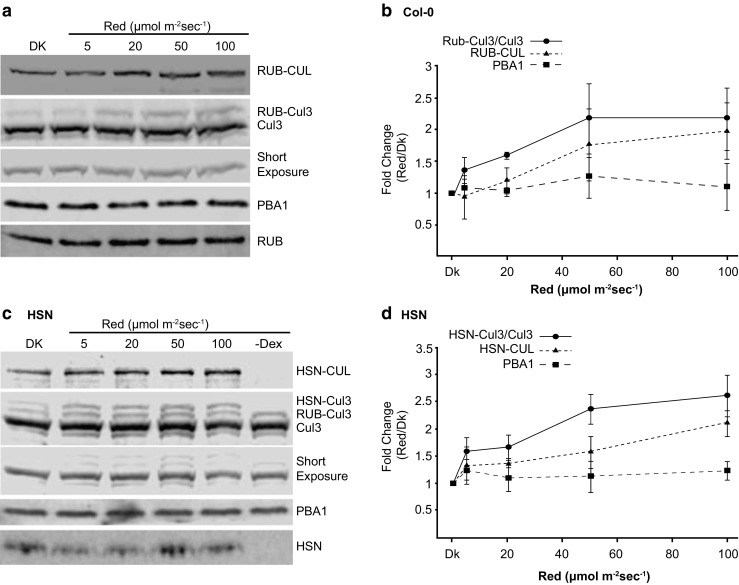
The RUBylation level of Cul3 and total CUL increases with increasing R intensity. **a, c** Immunoblot analysis of total protein extracts from 4-day-old Col-0 or HSN seedlings grown in Dk or R (5, 20, 50, and 100 μMol m^−2^ s^−1^). HSN–Dex seedlings were grown in R (50 μMol m^−2^ s^−1^). Total RUB and RUB-CUL or HSN and HSN-CUL were detected with anti-Nedd8 or anti-HA antibody respectively. Cul3 was detected with anti-Cul3 antibody. PBA1 was used as a loading control. The short exposure shows the relative levels of unmodified Cul3. **b**, **d** Quantification of the relative change in band intensity in R vs Dk of RUB-CUL, HSN-CUL, or RUB-Cul3/Cul3 displayed as a ratio of R/Dk. Error bars represent standard deviation (*n* = 3)

The RUBylation levels of AtCul3 in Col-0 and HSN showed substantial differences, with W and R significantly increasing compared to D controls. However, B did not significantly affect the RUBylation levels of Cul3 (Fig. 3). To understand if light intensity affects RUBylation levels, we assessed the RUB-Cul3 and total RUB-CUL in Col-0 and HSN exposed to various R intensities. We see that even in just 5 μMol m^−2^ s^−1^ R, there is an increased level of RUB-Cul3 and RUB-CUL compared to Dk controls in both Col-0 and HSN, and it generally continues to increase as the R intensity increases (Fig. 4). The control (PBA1) remained at similar levels in Dk and R treated samples. Together, these data suggest that W and R promote the RUBylation of Cul3, and this may be partially responsible for the total increase in RUB-CUL detected in W and R.

**Fig. 5:**
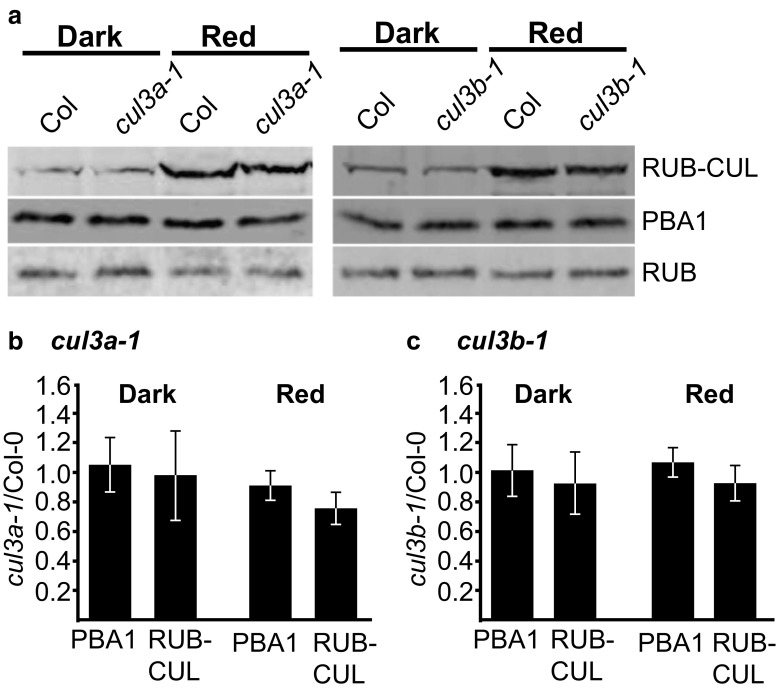
*cul3* mutants do not show significantly lower levels of RUB-CUL in R. **a** Immunoblot analysis of total protein extracts from 4-day-old Col-0, *cul3a-1*, and *cul3b-1* seedlings grown in Dk or R (50 μMol m^−2^ s^−1^). Total RUB and RUB-CUL were detected with anti-Nedd8 antibody. PBA1 was used as a loading control. **b**, **c** Quantification of the relative change in band intensity in *cul3a-1* or *cul3b-1* vs Col-0 for PBA1 or RUB-Cul displayed as a ratio of *cul3*/Col-0 in Dk or R. Error bars represents standard deviation (*n* = 3)

Since RUBylation of Cul3, but not Cul1, increases in response to W and R, we wanted to determine the relative extent that Cul3a and Cul3b contribute to the total increase of RUB-CUL in R light conditions. To do this, we assess the total RUB-CUL in *cul3a-1* and *cul3b-1* mutants using anti-N8 antibodies which do not discriminate between the different forms of RUB-Cul (Cul1, 2, 3, and 4). Both *cul3* mutants (*cul3a-1* and *cul3b-1*) did have slightly lower levels of RUB-CUL in R (Fig. 5a). Quantification of the band intensities revealed that *cul3a-1* was on average, 77% of Col-0 for RUB-CUL in R, while *cul3b-1* was 91% of Col-0 in R (Fig. 5b, c). However, these numbers were not significantly different than the changes in PBA1 in R between *cul3* mutants and Col-0 based on our experiments (*p* = 0.09 and 0.19 for *cul3a-1* and *cul3b-1*, respectively) (Fig. 5b, c). Perhaps a more significant difference could be found if both Cul3a and Cul3b were missing; however, the double mutant (*cul3a-1 cul3b-1*) is embryo lethal in *Arabidopsis*.

### Red Light Induces RUBylation of Cul3 in a Phytochrome B Dependent Manner

**Fig. 6:**
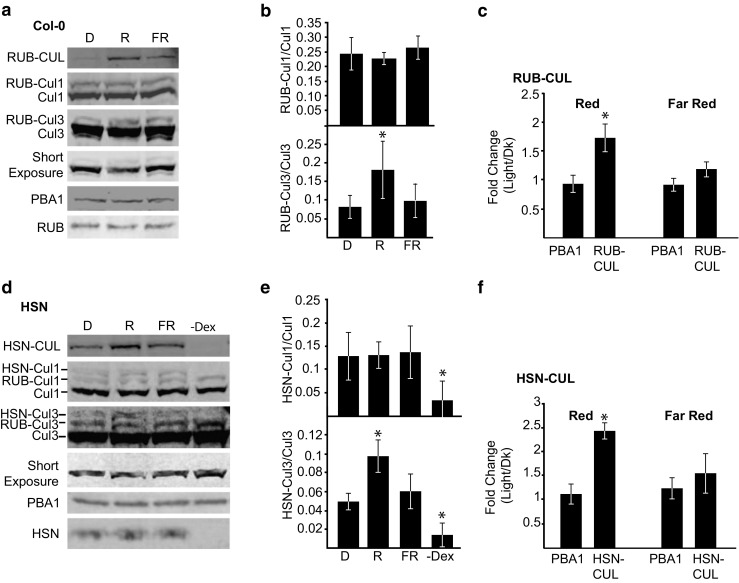
The RUBylation level of Cul3, but not Cul1 increases in R, but not FR light. **a**, **d** Immunoblot analysis of total protein extracts from 4-day-old seedlings grown in, Dk, R (50 μMol m^−2^ s^−1^) and FR (10 μMol m^−2^ s^−1^). HSN–Dex seedlings were grown in R. Total RUB and RUB-CUL or HSN and HSN-CUL were detected with anti-Nedd8 or anti-HA antibody respectively. Cul1 and 3 were detected with anti-Cul1 or -Cul3 antibody respectively. PBA1 was used as a loading control. The short exposure shows the relative levels of unmodified Cul3. **b**, **e** Quantification of RUB-Cul or HSN-Cul levels displayed as a ratio of RUB-Cul or HSN-Cul to unmodified Cul levels. Asterisks identify significant differences between D and light samples (*p* ≤ 0.05, Student’s *T* test). **c**, **f** Quantification of the relative change in band intensity in light (R or FR) vs Dk for PBA1, RUB-CUL, and HSN-CUL displayed as a ratio of light (R or FR)/Dk. Asterisks identify significant differences in the fold change between PBA1 and the RUB/HSN-CUL (*p* ≤ 0.05, Student’s *T* test). Error bars represents standard deviation (*n* = 3)

Plants detect R and FR through the phytochrome (phy) photoreceptor family, with phyB occupying the dominant role in the R response pathway. To determine if the RUBylation changes in R are dependent upon the activity of the phyB family, RUB-CUL levels were assessed in seedlings grown in R and FR, which activate and deactivate the phys respectively (Rockwell et al. 2006). For RUB-CUL and HSN-CUL, we saw an increase (1.7- and 2.5-fold) in R compared to Dk controls (Fig. 6). In addition, seedlings growing in FR displayed a small increase in the average levels of total RUB-CUL or HSN-CUL compared to Dk in both Col-0 and HSN; however, the differences were not significant compared to the PBA1 controls in our experiments (*p* = 0.051 and 0.48 for Col-0 and HSN, respectively) (Fig. 6c, f). When assessing the RUBylation levels of individual Culs, the RUBylation levels of Cul1 were similar in seedlings grown in Dk, R, and FR in both Col-0 and HSN. However, RUB-Cul3 levels increased significantly in R, but not in FR in both Col-0 and HSN (Fig. 6).

**Fig. 7:**
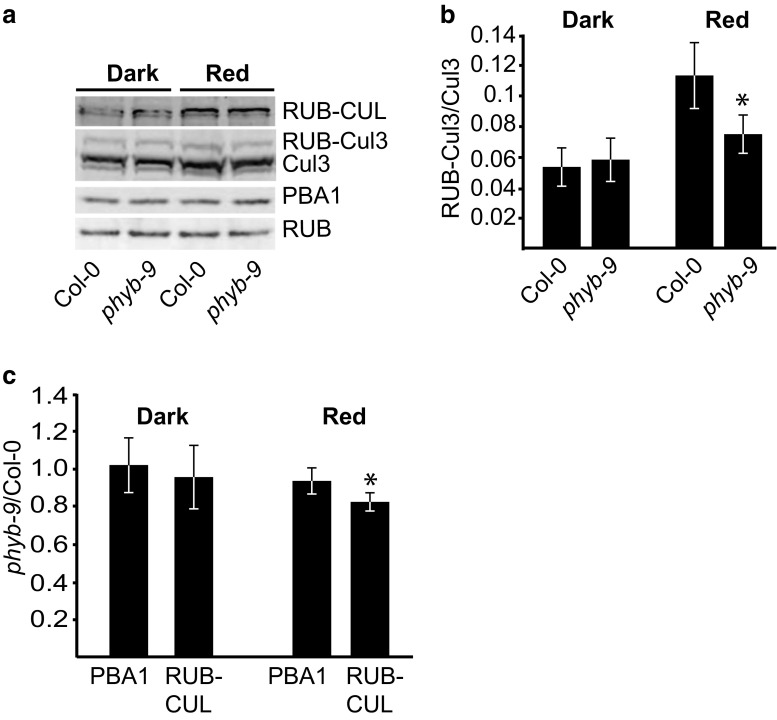
The RUBylation levels of Cul3 in R are partially dependent on active phyB. **a** Immunoblot analysis of total protein extracts from 4-day-old Col-0 and *phyB-9* seedlings grown in D and R (50 μMol m^−2^ s^−1^). Total RUB and RUB-CUL were detected with anti-Nedd8 antibody, respectively. Cul3 was detected with anti-Cul3 antibody. PBA1 was used as a loading control. **b** Quantification of RUB-Cul3 levels displayed as a ratio of RUB-Cul3 to unmodified Cul3 levels. Asterisks identify significant differences between D and light samples (*p* ≤ 0.05, Student’s *T* test). **c** Quantification of the relative change in band intensity in *phyb-9* vs Col-0 for PBA1 or RUB-Cul displayed as a ratio of *phyB-9*/Col-0 in Dk or R. Asterisks identify significant differences in the fold change between PBA1 and the RUB-CUL (*p* ≤ 0.05, Student’s *T* test). Error bars represents standard deviation (*n* = 3)

Analysis of RUB-Cul3 levels in the *phyB-9* mutant, which is R hyposensitive due to an absence of detectable phyB protein (Reed et al. 1993), reveals significantly lower levels of RUB-Cul3 compared to Col-0 in seedlings grown in 50 μMol m^−2^ s^−1^ R (Fig. 7a, b). When comparing the total RUB-CUL levels, we found that *phyb-9* was 82% that of the Col-0 levels in R, and this was significantly different than the changes in PBA1 between *phyB-9* and Col-0 (Fig. 5a, c). PhyB is the most dominant phytochrome for R responses; however, it works in conjunction with four other phytochrome family members (PhyA, C, D, and E) to sense R and FR light. Therefore, there is still some R signaling that is occurring in the *phyB-9* mutants, which may account for the smaller changes in RUB-Cul levels than we might expect if the plant was completely R insensitive. Taken together, these findings suggest that the activation of the phyB family plays a role in directing an increase in overall levels of Cul3 modification by RUB.

## Discussion

In an attempt to understand how E3 ligase activities may change in response to light, we assessed the overall RUBylation levels of CULs in different light conditions in *Arabidopsis thaliana*. We found that overall activation of all CUL-based E3 ligases, as assessed by RUBylation levels, is increased in response to long-term exposure to light. Intriguingly, W caused the greatest increase in RUBylation, R had modest increases, and B had little effect (Fig. 1).

What could drive this increase in RUBylation in specific light conditions? In vitro experiments on Human Cul1 and Nedd8 suggest that the availability of the substrate-bound adaptor promotes the formation of Neddylated-Cul1 by inhibiting the binding of CAND1 to CUL and preventing the activity of the CSN (Bornstein et al. 2006; Schmidt et al. 2009; Emberley et al. 2012). This model of activation for N8-CUL may explain the differences seen in our experiments. As a result of the immense changes that happen upon transition from dark to light, light-grown seedlings may contain more substrate that is available to be degraded compared to dark grown seedlings and therefore develop increased levels of RUB-CUL as a result. There are numerous examples of proteins in *Arabidopsis* known to be expressed at high levels, which get degraded upon light exposure. The R/FR photoreceptor PhyA, which can make up to 1% of the total protein in dark-grown seedlings, has a measured half-life of < 1 h after exposure to R and is degraded by the Cul4^COP1^ complex (Clough and Vierstra 1997; Seo et al. 2004). EIN3, a transcription factor regulating ethylene responses, is quickly degraded in response to R by the Cul1^EBF1/2^ complex (Shi et al. 2016).

Yet, the role of most E3 ligases in plants remains to be determined. Only about 10% of the 863 CUL-based E3 ligases in *Arabidopsis* have been associated with a specific pathway within the plant, and even fewer have been shown to associate with a known substrate (Hua and Vierstra 2011). Although the various CUL-based E3 ligases likely associate with different substrates at various developmental timepoints, without knowing all the different E3 substrates of different CULs, and the availability of those substrates, we are not able to determine if substrate availability is what is driving these differences in RUBylation levels. The overall levels of RUBylation do not significantly increase in B (Fig. 1), even though rapid degradation of select proteins has been shown to occur in B, including Cry2, the B photoreceptor (Ahmad et al. 1998). However, the E3 ligase associated with Cry2 degradation is still unknown and may not be a CUL-based E3. Many CUL-based E3 ligases (NPH3, RPT2, ZTL, FKF1, LKP2, COP1) have been identified that affect B signaling responses. Yet, several of these have unknown substrates, and others (ZTL, COP1) are actively ubiquitylating their targets (CIB1 and HY5 respectively) in darkness, not B (Hua and Vierstra 2011). Thus, it is possible that the overall activity of CUL-based E3 ligases in dark and B may exist at equal rates and, by comparison, show no global increased RUBylation. However, we do detect RUBylation differences in some light conditions. R triggers the increased RUBylation of Cul3-, but not Cul1-based E3 ligases (Figs. 2, 3, 4, and 6). This could suggest that more substrate is available for ubiquitylation with Cul3-, rather than the Cul1-based E3 ligases in R. The phyB family (B-E) is degraded by the Cul3^LRBs^ in R (Christians et al. 2012; Ni et al. 2014), which would support this. However, CUL-based E3 ligases such as Cul1^EBF1/2^ are also active in R, yet do not increase RUBylation levels of Cul1 (Fig. 2) (Shi et al. 2016).

Although substrate availability may potentially account for the differences in RUBylation, many of the specific protein degradation examples given here happen in a timescale of minutes to hours and may not reflect the differences in RUBylation detected in our experiments with seedlings grown in continuous light for several days. Instead, our results may indicate a sustained increase in overall activity of CULs, rather than an increased amount of degradation of a few individual substrate proteins at a specific point in development. This could be caused by several mechanisms. Since Neddylated-Cul1 and Neddylated-Cul3 have themselves been shown to rapidly degrade in human cells (Wu et al. 2005), perhaps there are differences in degradation of RUBylated CULs in different conditions in plants. RUB may be conjugated to CULs more efficiently by the conjugation pathway or perhaps the activity of RUB regulators like CANDI or the CSN is altered in response to different light conditions.

More experiments are needed to expand on these findings and determine the impact they have in plant growth and development. In particular, Cul4 and Cul2 were not assessed in any of these experiments, even though they may play a large role in degradation of substrates associated with light signaling, such as Cul4^COP1^. Cul3b only represented a small fraction of the total Cul3 in our experiments, and a more detailed analysis of this protein should be completed. In addition, there may be many more conditions, including hormone responses, biotic and abiotic stresses such as pathogen defense or drought, which may elicit similar changes in RUB-CUL levels. Such experiments may identify the extent plants control their E3 ligase activities to direct growth and development in various conditions in a more precise manner.
